# Intra-and inter-observer variability of point of care ultrasound measurements to evaluate hemodynamic parameters in healthy volunteers

**DOI:** 10.1186/s13089-023-00322-9

**Published:** 2023-05-05

**Authors:** Deborah Blanca, Esther C. Schwarz, Tycho Joan Olgers, Ewoud ter Avest, Nasim Azizi, Hjalmar R. Bouma, Jan Cornelis Ter Maaten

**Affiliations:** 1 Department of Internal Medicine, University of Groningen, University Medical Center Groningen, Groningen, The Netherlands; 2 Department of Internal Medicine, Ospedale Maggiore Policlinico, Università Degli Studi di Milano, Milan, Italy; 3 Department of Emergency Medicine, University Medical Centre Groningen, Groningen, The Netherlands; 4 Department of Clinical Pharmacy and Pharmacology, University of Groningen, University Medical Center Groningen, Groningen, The Netherlands

**Keywords:** Point of care ultrasound, Intra-observer variability, Inter-observer reliability, Repeatability, Reproducibility, Acute care

## Abstract

**Background:**

Point-of-care ultrasound (POCUS) is a valuable tool for assessing the hemodynamic status of acute patients. Even though POCUS often uses a qualitative approach, quantitative measurements have potential advantages in evaluating hemodynamic status. Several quantitative ultrasound parameters can be used to assess the hemodynamic status and cardiac function. However, only limited data on the feasibility and reliability of the quantitative hemodynamic measurements in the point-of-care setting are available. This study investigated the intra- and inter-observer variability of PoCUS measurements of quantitative hemodynamic parameters in healthy volunteers.

**Methods:**

In this prospective observational study, three sonographers performed three repeated measurements of eight different hemodynamic parameters in healthy subjects. An expert panel of two experienced sonographers evaluated the images’ quality. The repeatability (intra-observer variability) was determined by calculating the coefficient of variation (CV) between the separate measurements for each observer. The reproducibility (inter-observer variability) was assessed by determining the intra-class correlation coefficient (ICC).

**Results:**

32 subjects were included in this study, on whom, in total, 1502 images were obtained for analysis. All parameters were in a normal physiological range. Stroke volume (SV), cardiac output (CO), and inferior vena cava diameter (IVC-D) showed high repeatability (CV under 10%) and substantial reproducibility (ICC 0.61–0.80). The other parameters had only moderate repeatability and reproducibility.

**Conclusions:**

We demonstrated good inter-observer reproducibility and good intra-observer repeatability for CO, SV and IVC-D taken in healthy subjects by emergency care physicians.

**Supplementary Information:**

The online version contains supplementary material available at 10.1186/s13089-023-00322-9.

## Background

Assessment of the hemodynamic status is important in emergency medicine to predict which patients may benefit from volume resuscitation adequately or may develop adverse outcomes [1]. History taking and physical examination are often insufficient to determine hemodynamic status, and they suffer from significant limitations [2]. Over the last few years, point-of-care ultrasound (PoCUS) has shown to be a valuable tool for promptly obtaining more detailed information about the hemodynamic status of patients at the emergency department (ED), especially in patients with shock [3, 4]. POCUS uses a qualitative approach to perform hemodynamic measurements, often as part of a pre-defined algorithm such as Rapid Ultrasound for Shock and Hypotension (RUSH) protocol [4] and Echo Guided Life Support (EGLS) protocol [5]. Nevertheless, inexperienced practitioners must be aware of some common misinterpretations related to this approach that may lead to wrong decisions at the bedside [6].

Quantitative measurements, however, have potential advantages over qualitative evaluation of hemodynamic status. Several quantitative ultrasound parameters exist to evaluate the hemodynamic status and cardiac function: Overall cardiac function can be assessed by measuring cardiac output (CO), carotid blood flow (CBF) [1, 11], or stroke volume (SV) [7] or by quantification of individual components of CO such as cardiac contractility. E-Point septal separation (EPSS) [8] and Mitral annulus plane systolic excursion (MAPSE) [9] are commonly used to assess left ventricular systolic function; the Tricuspid annulus plane systolic excursion (TAPSE) [10] is used for the assessment of right ventricular systolic function. Hemodynamic status can be evaluated by measuring Inferior Vena Cava Diameter (IVC-D) and Collapsibility Index (IVC-CI). All aforementioned quantitative POCUS parameters are noninvasive and rapidly repeatable; using these parameters, singularly or in combination, can play a role in patient evaluation and hemodynamic status detection in patients in the emergency setting [11].

So far, only limited data are available on the feasibility of the quantitative hemodynamic measurements in the point-of-care setting [12, 13] and data about the reliability of the various hemodynamic measurements are sparse [14]. Moreover, those studies were focused on ultrasound image reinterpretations by a different observer but not on the operator’s repeatability, and generally, they considered only one or a few ultrasound parameters [12, 14, 15]. Furthermore, it is unknown whether specialists in acute internal medicine can reliably perform quantitative POCUS measurements to assess cardiac function and hemodynamic status.

We have performed a prospective observational study to simultaneously investigate the intra- and inter-observer variability of several POCUS quantitative hemodynamic parameters. POCUS measurements were taken in healthy volunteers by the same sonographers.

## Materials and methods

### Study design

This prospective observational study was conducted between 8th September 2020 and 25th January 2021 at the University Medical Center Groningen (UMCG) to investigate the intra and inter-observer variability of various quantitative hemodynamic parameters obtained by POCUS in healthy volunteers by specialists in acute internal medicine. This study has been approved by the central ethical commission (CTc UMCG), registration number: 202000917.

### Subjects

The study population consisted of 32 healthy adult volunteers. Subjects were eligible for participation when they were aged > 18 and had no previously known cardiovascular medical conditions. Patients were excluded if they were unable to tolerate a supine position for the duration of the ultrasound examination or when they were pregnant. Written informed consent was obtained from all volunteers.

### Measurements

Three different specialists in acute internal medicine examined the included subjects. All three sonographers have several years of experience in qualitative POCUS ultrasound with at least Entrustable Professional Activity (EPA- level) 4 [16]. This means they have performed at least 25 ultrasound exams for each specific POCUS application, e.g., at least 25 POCUS echocardiography exams and 25 IVC measurements. However, they had little knowledge of the use of doppler, and they never made the quantitative measurements of the parameters used in this study (except for the inferior vena cava (IVC) measurements), making them relatively novices. Before starting the study, each sonographer received instructions about making POCUS measurements involved in the research and a two-hour training session. This consisted of explaining how to make the proper calculations using the ultrasound machine program and in a training moment where the sonographers could practice each ultrasound parameter execution on several volunteers.

For the study, each investigator measured eight different hemodynamic parameters and repeated each measurement three times (starting over and changing the probe position after each measurement). At least two investigators examined all subjects. All measurements in individual subjects were conducted within 1.5 h by the different investigators to ensure similar conditions and avoid changes in hydration status or other hemodynamic factors. The investigators did not see each other performing the measurements.

### Data collection

All examinations were performed with the GE Venue R1 (General Electric Healthcare, Chicago, USA), equipped with automatic tools to measure hemodynamic functions, including the cardiac output, the inferior vena cava collapsibility index and diameter, and the carotid blood flow (CBF).

In our study, we measured LVOT-D, EPSS, MAPSE, and TAPSE manually. For the other measurements (LVOT-VTI, IVC-D, rCBF–D, and rCBF–VTI), automatic tools on the ultrasound machine were used. From the eight different measurements described above, additional four parameters were calculated automatically from the GE Venue R1 (SV, CO, IVC-CI and rCBF). A total of twelve ultrasound parameters were registered, as described in Additional file 1: Table S1. To standardize the exams, an ultrasound protocol (Additional file 1: Table S2) based on several ultrasound guidelines was used [4, 17].

The data were stored anonymously on the UMCG research platform; the images were reviewed and analyzed with the MicroDicom DICOM-viewer. A quality review of the images obtained was performed, before analysis took place. Two blinded experienced sonographers evaluated the images following sonographic assessment criteria based on the standard of the American Society of Echocardiography [17]. If more than one of the three images of the same parameter of the same observer were of poor quality, the whole set of three measurements was excluded from further analysis.

### Primary endpoint

Evaluation of repeatability (intra-observer variability) and reproducibility (inter-observer variability) of each of the twelve individual quantitative hemodynamic POCUS measurements taken in healthy volunteers by acute physicians experts in qualitative POCUS evaluation underwent training for those specific quantitative POCUS measurements.

### Data analysis

Repeatability was determined by calculating the coefficient of variation (CV) between the separate measurements for each observer. We took the average of the CV across all models to measure overall repeatability. Based on Kirkwood et al. we considered a CV of 20% as high dispersion of data, thus a high variability, and a CV of 10% as a low dispersion of data, thus a low variability, whereby the range between the two was considered moderate [18].

Reproducibility was assessed by determining the intra-class correlation coefficient (ICC) [16] using a two-way random-effects model. To facilitate interpretation, we applied the Portney and Watkins classification [19]. The normality of data was tested using the Shapiro–Wilk test before running the model.

Statistical analysis was performed using Microsoft Excel 2010 and SPSS Statistic version 23 (IBM Corp.Armonk, USA).

## Results

### Ultrasound image acquisition and selection

A total of 32 subjects participated in the study. There were 18 (56%) females and 14 (44%) males, with a mean (SD) age of 23 (2.96) years.

The three sonographers examined 30, 27, and 28 volunteers, respectively. Twenty-one participants received ultrasound evaluations from all three observers. A total of 595 sets of images (1785 in total) were taken. The number of sets of images available for each parameter and observer is given in Additional file 1: Table S3.

**Fig. 1:**
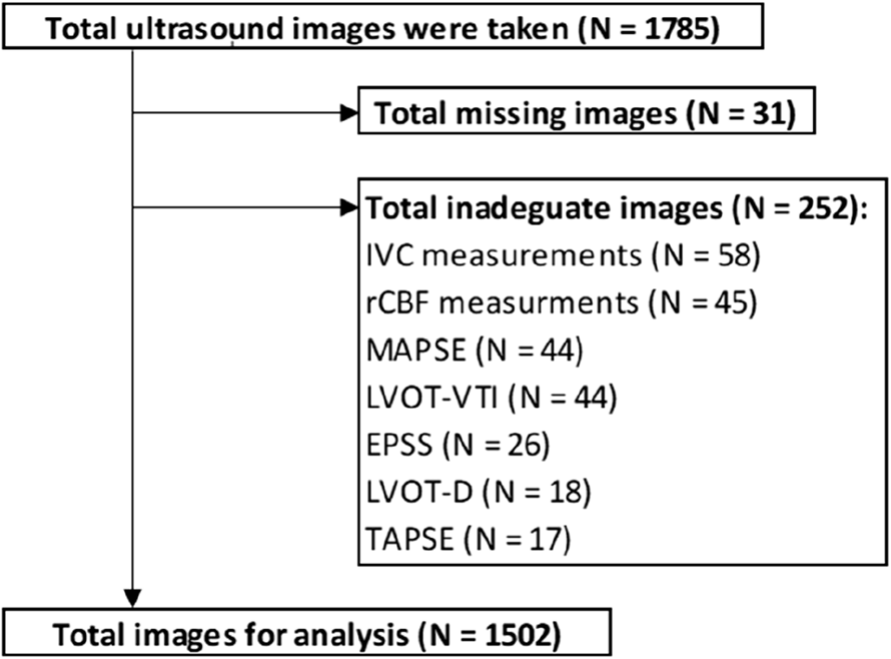
Flow chart of inclusion and exclusion of all patients

Of the total 1785 ultrasound images taken, 31 images were missing, mainly due to storage issues of the GE Venue R1 apparatus, and a total of 252 (14%) images did not fulfill the quality assessment criteria and were excluded from the analysis. We included a total of 1502 images for analysis (Fig. 1).

The main reason for excluding IVC-D and IVC-CI images was the presence of artifacts in the calculation time interval (auto measurements). Most MAPSE images were excluded due to the incorrect obliqueness of the image (M Mode and cardiac image); the others were excluded due to the wrong alignment of the M Mode cursor along the mitral lateral annulus and misalignment of the septum. CBF measurement images were excluded mainly due to inadequate tracing of the VTI lines, followed by wrong sample gate placement and the obliqueness of the vessel walls. Moreover, lastly, we excluded LVOT-VTI images because the sample gate cursor placement was incorrect or due to the bad quality of the VTI spectral tracing.

Results of image quality assessment per parameter and observer, missing images, and total images for analysis are summarized in Additional file 1: Table S4.

### Hemodynamic parameters

Baseline values (mean ± SD) of the hemodynamic parameters measured with POCUS stratified by observers are represented in Table 1. As expected for healthy volunteers, all parameters were in a normal physiological range.

**Table 1:** Baseline characteristics (mean ± SD) parameter

Parameter*	Baseline characteristics (mean ± SD) parameter				
	Observer 1 (*N* = 30)	Observer 2 (*N* = 27)	Observer 3 (*N* = 28)	Mean (*N* = 32)	Normale range
Measured					
LVOT-D (mm)	21.0 ± 0.5	20.6 ± 0.6	20.9 ± 0.4	20.8 ± 0.5	20–25
LVOT-VTI (cm)	21.6 ± 1.2	20.7 ± 1.3	18.5 ± 1.2	20.3 ± 1.2	> 18
EPSS (mm)	3.5 ± 0.7	4.9 ± 0.9	2.9 ± 0.5	3.7 ± 0.7	< 7
MAPSE (mm)	18.1 ± 1.1	18.0 ± 1.9	17.4 ± 1.7	17.8 ± 1.6	> 10
TAPSE (mm)	26.9 ± 1.3	26.9 ± 2.2	25.2 ± 1.6	26.3 ± 1.7	> 16
IVC-D (mm)	20.0 ± 0.8	18.8 ± 0.8	20.3 ± 0.9	19.7 ± 0.9	< 21
rCBF-D (mm)	6.3 ± 0.3	6.1 ± 0.5	6.1 ± 0.3	6.2 ± 0.4	
rCBF-VTI (cm)	26.0 ± 2.5	19.5 ± 2.2	20.7 ± 2.3	22.0 ± 2.3	
Calculated					
CO (l/min)	4.5 ± 0.2	4.5 ± 0.3	4.2 ± 0.3	4.4 ± 0.3	4.4–5.0
SV (ml)	68.7 ± 3.6	66.5 ± 3.8	63.1 ± 4.5	66.1 ± 4.0	60–100
IVC-CI (%)	34.6 ± 3.5	43.5 ± 8.0	34.9 ± 6.6	37.7 ± 6.0	< 50
rCBF (mL/min)	478.5 ± 53.2	346.7 ± 66.5	357.4 ± 51.7	394.2 ± 57.2	

*LVOT-D* left ventricular outflow tract diameter, *LVOT VTI* left ventricular outflow tract velocity time integral, *EPSS* E point septal separation, *MAPSE* mitral annular plane systolic excursion, *TAPSE* tricuspid annulus plane systolic excursion, *IVC-D* inferior vena cava diameter, *rCBF-D* right Carotid blood flow diameter, *rCBF-VTI* right Carotid blood flow velocity–time integral, *CO* Cardiac output, *SV* stroke volume, *IVC-CI* inferior vena cava collapsibility index, *rCBF* right Carotid blood flow

*Each parameter was taken three times in every patient (3 × N. of volunteers screened)

### Repeatability

The results related to intra-observer variability are summarized in Table 2.

**Table 2:** Intra-researcher variability for each POCUS parameter

Parameter	Intra-observer variability based on mean CV in %			
	Observer 1 (*N* = 540)	Observer 2 (*N* = 469)	Observer 3 (*N* = 493)	Mean (*N* = 1502)
Measured				
LVOT-D	2.54	2.96	2.18	2.56
LVOT-VTI	5.66	6.22	7.34	6.41
EPSS	23.07	19.74	34.25	25.69
MAPSE	6.25	10.46	9.97	8.89
TAPSE	4.86	8.26	6.18	6.43
IVC-D	4.45	4.22	4.82	4.50
rCBF-D	4.91	7.81	4.82	5.85
rCBF-VTI	10.09	10.72	10.89	10.57
Calculated				
CO	5.48	6.24	7.26	6.33
SV	5.50	6.01	7.68	6.39
IVC-CI	12.25	19.63	18.46	16.78
rCBF	11.63	18.91	14.03	14.86

*CV* coefficient of variation, *LVOT-D* left ventricular outflow tract diameter, *LVOT VTI* left ventricular outflow tract velocity time integral, *EPSS* E point septal separation, *MAPSE* mitral annular plane systolic excursion, *TAPSE* tricuspid annulus plane systolic excursion, *IVC-D* inferior vena cava diameter, *rCBF-D* right Carotid blood flow diameter, *rCBF-VTI* right Carotid blood flow velocity–time integral, *CO* Cardiac output, *SV* stroke volume, *IVC-CI* inferior vena cava collapsibility index, *rCBF* right Carotid blood flow

The LVOT-D parameter showed the lowest intra-observer variability (2.54%). The IVC-D, rCBF-D, CO, SV, LVOT-VTI, TAPSE, and MAPSE all had a mean CV under 10% (high repeatability). The rCBF (and rCBF-VTI) and the IVC-CI demonstrated moderate repeatability (intra-observer variability between 10 and 20%). Only the EPSS showed a high variance with a mean CV higher than 20% (low repeatability). The variables in increasing order of CV are shown in Additional file 1: Graph 1.

### Reproducibility

The inter-observer variability, calculated for those patients for whom full sets of images were available for all three examiners, is represented in Table 3. Data about the ICC based on the comparison between operators are summarized in Additional file 1: Table S5.

**Table 3:** Inter-researcher variability: ICC based on all three examiners’ full sets of images, with the 95% confidence interval and the correlating sample size number of volunteers

Parameter	ICC	95% Confidence interval		Sample size (*N* = 21)
		Lower border	Upper border	
Measured				
LVOT-D	0.518	0.237	0.763	17
LVOT-VTI	0.427	0.098	0.739	13
EPSS	0.427	0.098	0.739	13
MAPSE	0.409	0.087	0.714	15
TAPSE	0.303	0.028	0.605	18
IVC-D	0.655	0.295	0.876	12
rCBF-D	0.302	− 0.054	0.682	12
rCBF-VTI	0.152	− 0.071	0.511	12
Calculated				
CO	0.621	0.309	0.848	13
SV	0.693	0.414	0.881	13
IVC-CI	0.472	0.135	0.777	12
rCBF	0.148	− 0.052	0.489	12

*ICC* intra-class correlation coefficient, *LVOT-D* left ventricular outflow tract diameter, *LVOT VTI* left ventricular outflow tract velocity time integral, *EPSS* e point septal separation, *MAPSE* mitral annular plane systolic excursion, *TAPSE* tricuspid annulus plane systolic excursion, *IVC-D* inferior vena cava diameter, *rCBF-D* right Carotid blood flow diameter, *rCBF-VTI* right Carotid blood flow velocity–time integral, *CO* cardiac output, *SV* stroke volume, *IVC-CI* inferior vena cava collapsibility index, *rCBF* right Carotid blood flow

A substantial reproducibility (ICC 0.61–0.80) was found for SV, CO, and IVC-D. A moderate reproducibility (ICC 0.41–0.60) was seen in MAPSE, LVOT-VTI and LVOT-D, IVC-CI, and EPSS. However, rCBF and TAPSE showed fair reproducibility (ICC 0.21–0.40).

### Integrating repeatability and reproducibility of hemodynamic parameters

To assess which hemodynamic function parameter showed the most reliable outcome for the repeatability per observer and reproducibility between observers, we compared intra-observer variability (CV) with inter-observer variability (ICC). CO, SV and IVC-D had a low intra-observer variability and substantial reproducibility, implying that of all examined parameters, they have the best balance between CV and ICC.

## Discussion

This study showed good inter-observer reproducibility and good intra-observer repeatability for CO, SV and IVC diameter measurements.

Ultrasound image acquisition and interpretation are operator dependent [20]; only a few studies describe intra- and inter-observer variabilities for hemodynamic ultrasound parameters. They are mainly based on the reinterpretation of the same ultrasound images by a different observer, but not on the same operator's repeatability. They generally focus on only one or a few ultrasound parameters [12, 14, 15]. This is in contrast to our study, which also assesses the repeatability and reproducibility of POCUS measurements. Moreover, to determine the inter-observer reproducibility, we used the intraclass correlation coefficient, which allows the analyses of both the degree of correlation and the level of agreement of the measurements made by the different observers. Furthermore, we have analyzed the feasibility of simultaneously performing both cardiac function and hemodynamic status parameters. Previous feasibility studies mainly focused on only one parameter [12, 13]. Thus, this is the first study where intra- and inter-observer variabilities for hemodynamic ultrasound parameters were studied simultaneously.

Of all parameters regarding cardiac function, stroke volume and cardiac output showed the best test characteristics. This seems to be unexpected because the measurement of these parameters presents certain challenges: any inaccuracies in the measurement of LVOT diameter are taken to the second power in the continuity equation [21], and inconsistent placement of the pulsed Doppler sample volume in the LVOT is a consistent source of error [22]. Previous studies suggested that cardiac output can only be reliably measured by more experienced observers [23]. In our study, the sonographers already had experience performing qualitative cardiac function ultrasound, which may explain why a short introduction training was enough. Also, cardiac output was automatically determined by the GE Venue R1 machine using an automated VTI tracing system. This is in line with other feasibility studies showing the increasing quality of ultrasound images with an increasing level of training [13]. Hence, even though technical difficulties are described in the literature, we obtained reliable CO and SV measurements after a short training program. As already known in the literature, automatic tools correlate closely with manual measurements for LVOT-VTI measurements [24]; the vantage we could have was that the automatic method could allow realizing measurements within a much shorter time than the standard manual tracing method [25]. We think we could obtain the same result by calculating the LVOT-D measurement manually, as we did, and also tracing the LVOT-VTI measurement manually. The automatic tool helped us trace the wave and make calculations (using the equation), but we did not have any advantage in angle alignment. The vantages we had were about saving time, making the process more executable in the emergency environment, and reducing the possibility of calculation errors.

We found a moderate reproducibility and a high exclusion rate of the MAPSE and the TAPSE ultrasound images, which we did not expect as these parameters are measured in only one ultrasound window and thereby allow brief examination. We revealed that obtaining MAPSE and TAPSE images for specialists in acute internal medicine might be more challenging than for more trained cardiac examiners like cardiologists. The differences in reliability between our result and the literature might be due to the difference in the study population. The previous studies analyzed bigger sample sizes and a more diverse study population, with patients with both physiological and pathological values. It can be argued that the diversity in outcomes for the individual patients, in its extremity, led to a higher correlation and agreement of data and, therefore, better reproducibility [26, 27].

We found a high exclusion rate for the CBF measurements due to inadequate tracing of the automatic VTI line functions. The intra-observer variability of the CBF was only moderate, in line with other studies demonstrating a large range of reproducibility [28]. Some explanations for this difference in reproducibility might be the difference in the location of the measurement [29] and the physiological carotid artery diameter changing during systole and diastole [30]. Because the VTI curve and the tracing were frequently not aligned, it is possible that fewer images would have been excluded by manually tracing the VTI.

Identifying the IVC diameter and collapsibility is part of the essential ultrasound examination at the ED [31, 32] to assess the hemodynamic status and guide fluid resuscitation. Previous studies have shown that the IVC-CI and IVC-D ultrasound measurements can easily be performed with minimal training [33]. Although our intra-observer variability and inter-observer reproducibility for the IVC-D were both good, IVC-CI intra-observer variability and inter-observer reliability were only moderate. This corresponds with the findings of previous studies [34]. We think that the reliability of the IVC measurements might have been influenced by the automatic trace of the vessel in M-mode and partially explains the high number of excluded images. We suppose IVC-CI measurements showed more variation than IVC-D measurements due to a slight variation in breathing. On the other hand, the automated tracing of the IVC-CI measurements could be more vulnerable due to variance of interest (because it is calculated from two measurements) than the IVC-D measurements, which are only affected by natural variation (unwanted variance). We don’t think there was a difference between automated and manual tracing of IVC diameter. The automatic tool traced the vessel; manual tracing would give the same result. The vantage was that the automatic tool eliminated variation due to human error when measuring IVC.

There are limitations to our study. First, it has a relatively small sample size. We might have found different results if we had analyzed a larger population. Moreover, it was only sometimes possible for all three observers to examine each participant due to scheduling difficulties, so only some subjects received measurements from all three observers. Second, this is a feasibility study conducted on healthy volunteers, and practicability may differ in real patients in acute care settings: measurements in healthy subjects can be more accessible because they can maintain the asking posture and guarantee optimal preparation. Moreover, the measurements would be in the physiological range, with a low variance rate. Third, a measurement bias due to the observer bias [35] may have occurred if the individual observers tried to match measurements to their previous examinations, considering they were aware that their measurements were being studied [36]. Fourth, we used automated measuring tools, which the ultrasound machine we have in our emergency department was equipped with; but those functions are only readily available in some emergency departments. In addition, using manual measurements (instead of auto tools) could make the measurement acquisition process longer and, therefore, more challenging to measure in the field of an emergency department. Fifth, the three sonographers have several years of experience in qualitative POCUS ultrasound and could perform quantitative measurements after a brief instruction and a two-hour training session. If more limited experience sonographers had performed the same POCUS measurements, the study would have given other results. Sixth, ICC was calculated only when reliable measurements could be obtained. Because the ICC depends on N: the lower the N, the higher the ICC when the mean and standard deviation remain the same. By selecting only patients in whom both base parameters were considered acceptable (e.g., for CO and SV), we had a lower N. This means that these numbers are based on a slightly different data set, which impacts the ICC due to relatively lower numbers and can contribute to an artificially higher ICC.

## Conclusions

We demonstrate good inter-observer reproducibility and intra-observer repeatability for CO, SV and IVC-D measurements by those specialists in acute internal medicine with basic ultrasound knowledge that underwent a specific limited training program. Those measurements have relevance for evaluating the hemodynamic status in healthy volunteers. Future studies should validate the observed findings in hemodynamically unstable patients.

## Supplementary Information

**Additional file 1: Supplementary Table 1:** Description of the methods used to acquire the parameters of interest and the procedure for measuring and automatically calculating them with GE Venue R1. **Supplementary Table 2:** Description of the ultrasound protocol used to acquire the parameters, based on the "Guidelines for performing a comprehensive transthoracic echocardiographic examination in adults: recommendations from the American Society of Echocardiography." **Supplementary Table 3:** Numbers of available image sets divided by each parameter and observer. **Supplementary Table 4:** Image quality assessment results divided by parameter and observer, missing images, and total images for analysis. **Supplementary Table 5:** Data about the ICC based on operator comparison.

## Data Availability

The datasets used and/or analyzed during the current study are available from the corresponding author on reasonable request.

## Abbreviations

AC: Angle of correction
CB: Carotid bulb
CC-A: Common carotid artery
CCA-D: Common carotid artery diameter
CBF: Carotid blood flow
CO: Cardiac output
CV: Coefficient of variation
CVP: Central venous pressure
ED: Emergency department
EPSS: E Point septal separation
FOCUS: Focus cardiac ultrasound
FV: Flow volume
ICC: Intraclass correlation coefficient
IVC: Inferior vena cava
IVC-CI: Inferior vena cava collapsibility index
IVC-D: Inferior vena cava diameter
IVC-M: Inferior vena cava measurements
LVEF: Left ventricular ejection fraction
LVOT: Left ventricular outflow tract
LVOT-D: Left ventricular outflow tract diameter
LVOT VTI: Left ventricular outflow tract velocity time integral
MAPSE: Mitral annular plane systolic excursion
M-Mode: Motion mode
PAC: Pulmonary artery catheter
POCUS: Point of care ultrasound
PS: Peak systolic velocity
PSLA: Parasternal long axis
RUSH: Rapid ultrasound for shock and hypotension
rCBF: Right carotid blood flow
RVOT: Right ventricular outflow tract
SV: Stroke volume
SVR: Systematic vascular resistance
TAMEAN: Time average mean velocity
TAMAX: Time average maximum velocity
TAPSE: Tricuspid annulus plane systolic excursion
TV: Tricuspid valve
VTI: Velocity time integral
