# Supplementary material for: Intra-and inter-observer variability of point of care ultrasound measurements to evaluate hemodynamic parameters in healthy volunteers
Source: Ultrasound J. 2023 May 5;15:22. doi: 10.1186/s13089-023-00322-9 (PMC10163179; doi:10.1186/s13089-023-00322-9)
Supplement: Supplementary file 1 — Additional file 1: Supplementary Table 1: Description of the methods used to acquire the parameters of interest and the procedure for measuring and automatically calculating them with GE Venue R1. Supplementary Table 2: Description of the ultrasound protocol used to acquire the parameters, based on the "Guidelines for performing a comprehensive transthoracic echocardiographic examination in adults: recommendations from the American Society of Echocardiography." Supplementary Table 3: Numbers of available image sets divided by each parameter and observer. Supplementary Table 4: Image quality assessment results divided by parameter and observer, missing images, and total images for analysis. Supplementary Table 5: Data about the ICC based on operator comparison. [file 13089_2023_322_MOESM1_ESM.docx]

## Appendix

### Supplementary Tables

#### Supplementary Table 1: Description of POCUS parameters obtaining procedure

| **Parameter** | **POCUS parameters obtaining procedure** |  |
| --- | --- | --- |
| **Measured** | |  |
| LVOT-D | Obtained from the parasternal long-axis ultrasound view (PSLA) with a phase-arrayed probe. Measured manually from endocardium to endocardium approximately 3 to 10 mm below the level of the aortic valve annulus during mid-systole. It was expressed in mm. |  |
|  |  |  |
|  |  |  |
|  |  |  |
| LVOT-VTI | Obtained in the apical five-chamber view (A5C) with a phase-arrayed probe. To take the measurements, the pulse wave doppler gate was positioned perpendicularly over the left ventricular outflow tract. The ultrasound machine automatically* calculated the velocity time index from the pulse wave doppler trace. I was measured in cm. |  |
|  |  |  |
|  |  |  |
|  |  |  |
| EPSS | Obtained in the PSLA with a phase-arrayed probe using M-Mode. The M-Mode beam was pointing through the most apical point of the mitral valve in early diastole at maximal excursion of the mitral valve leaflet. The EPSS was calculated manually by measuring between the septum and the most apical point of the mitral valve and measured in mm. |  |
|  |  |  |
|  |  |  |
|  |  |  |
| MAPSE | Obtained in the apical four-chamber view (A4C) with a phase-arrayed probe. The MAPSE was identified by measuring the distance manually from end-diastole to end-systole and measured in mm. It was assessed by recording the movement of the lateral mitral annulus between diastole and systole in M-Mode. |  |
|  |  |  |
|  |  |  |
|  |  |  |
| TAPSE | Obtained in the apical four-chamber view (A4C) with a phase-arrayed probe. The TAPSE was identified by measuring the distance manually from end-diastole to end-systole and measured in mm. It was assessed by recording the movement of the right lateral cardiac border through the tricuspid annulus between diastole and systole in M-Mode. |  |
|  |  |  |
|  |  |  |
|  |  |  |
| IVC-D | Obtained from the subxiphoid window with a phase-arrayed probe. The M-Mode sample gate was placed in the center of the vessel’s long-axis view, 2 cm from the right atrium. The GE Venue system measured automatically, at the same time, the maximal and minimal diameters of the IVC throughout one respiratory cycle by tracing the M Mode timeline (red lines in auto IVC – Mode). It was measured in mm. |  |
|  |  |  |
|  |  |  |
|  |  |  |
| rCBF-D | Obtained at the same level as the Doppler sample gate placement (taken in the same image stored), perpendicular to the vessel walls in the longitudinal view of the right common carotid artery. The ultrasound machine automatically calculated the carotid cross-sectional area (CCA-CSA). It was measured in mm. |  |
|  |  |  |
|  |  |  |
|  |  |  |
| rCBF-VTI | Obtained by placing the pulse wave Doppler sample gate 0,5 cm below the inferior margin of the carotid bulb at the center and perpendicular to the vessel, with an inclination angle of < 60°. The ultrasound machine automatically calculated the velocity time index from the pulse wave doppler trace. It was measured in cm. |  |
|  |  |  |
|  |  |  |
|  |  |  |
| **Calculated** | |  |
| CO | Automatically calculated by the ultrasound machine using the following formula. CO: LVOT-VTI x (½ x LVOT-D)2 x π) x HR (heart rate). Expressed in l/min. |  |
|  |  |  |
|  |  |  |
|  |  |  |
| SV | Automatically calculated by the ultrasound machine using the following formula. SV: LVOT-VTI x (½ x LVOT-D)2 x π). Expressed in ml. |  |
|  |  |  |
|  |  |  |
|  |  |  |
| IVC-CI | Automatically calculated by the ultrasound machine using maximal and minimal IVC diameters throughout one respiratory cycle. We have used the following formula: IVCCI=100x(1−IVCDmin/IVCDmax). Expressed in percentage. |  |
|  |  |  |
|  |  |  |
|  |  |  |
| CBF | Automatically calculated by the ultrasound machine by tracing the rCBF-VTI and measuring the CCA-CSA. The following formula is used: CBF (CM/MIN) = CCA-CSA x CCA-VTI. Calculated in mL/min. |  |
|  |  |  |
|  |  |  |
|  |  |  |

This table provides a description of the methods used to acquire the parameters of interest and the measurement and automated calculation procedure using GE Venue R1.

Legend: LVOT-D - Left ventricular outflow tract diameter; LVOT VTI - Left ventricular outflow tract velocity time integral; EPSS – E Point septal separation; MAPSE - Mitral annular plane systolic excursion; TAPSE – Tricuspid annulus plane systolic excursion; IVC-D - Inferior vena cava diameter; rCBF-D - right Carotid blood flow diameter; rCBF-VTI - right Carotid blood flow velocity-time integral; CO – Cardiac output; SV – Stroke volume; IVC-CI - Inferior vena cava collapsibility index; rCBF – right Carotid blood flow. * Automated measurements of LVOT-VTI of spectral Doppler correlate closely with manual measurements [25]; moreover, the automatic method could also allow realizing these measurements within a much shorter time than the standard manual tracing method [26]

####

#### Supplementary Table 2: Ultrasound protocol: probe, frequency, settings, patient position, ultrasound views.

| **Parameter** | **Probe** | **Frequency (MHz)** | **Settings** | **Patient position** | **Ultrasound views** |
| --- | --- | --- | --- | --- | --- |
| CO, SV, LVOT-D, LVOT-VTI | Phased array | 1.0 - 5.0 | Cardiac | Supine, head elevated by 20̊ | PSLA |
|  |  |  |  | Left lateral decubitus position | Apical five chamber |
| EPSS | Phased array | 1.0 - 5.0 | Cardiac | Supine | PSLA |
| MAPSE | Phased array | 1.0 - 5.0 | Cardiac | Left lateral decubitus position | Apical four chamber |
| TAPSE | Phased array | 1.0 - 5.0 | Cardiac | Left lateral decubitus position | Apical four chamber |
| IVC-CI, IVC-D | Phased array | 1.0 - 5.0 | Abdomen | Supine | Subxiphoid |
| rCB, rCBF-D, rCBF-VTI | Linear | 5.0 - 12.0 | Vascular access-Carotid | Supine, head rotated approximately 30° to the contralateral side | 5 mm below the inferior margin of the carotid bulb |

This table provides a description of the ultrasound protocol used to acquire the parameters, based on the "Guidelines for performing a comprehensive transthoracic echocardiographic examination in adults: recommendations from the American Society of Echocardiography".

Legend: LVOT-D - Left ventricular outflow tract diameter; LVOT VTI - Left ventricular outflow tract velocity time integral; EPSS – E Point septal separation; MAPSE - Mitral annular plane systolic excursion; TAPSE – Tricuspid annulus plane systolic excursion; IVC-D - Inferior vena cava diameter; rCBF-D - right Carotid blood flow diameter; rCBF-VTI - right Carotid blood flow velocity-time integral; CO – Cardiac output; SV – Stroke volume; IVC-CI - Inferior vena cava collapsibility index; rCBF – right Carotid blood flow.

#### Supplementary Table 3: Total amount of images taken and sets of images

| **Parameter** | **Total sets of images (N=32)** | | | |  |
| --- | --- | --- | --- | --- | --- |
|  |  |  |  |  |  |
|  | **Observer 1 (N=30)** | **Observer 2 (N=27)** | **Observer 3 (N=28)** | **All three observers (N=21)** |  |
| **Measured** |  |  |  |  |  |
| LVOT-D (cm) | 28 | 24 | 27 | 17 |  |
| LVOT-VTI (cm) | 28 | 25 | 20 | 13 |  |
| EPSS (mm) | 27 | 25 | 27 | 16 |  |
| MAPSE (mm) | 27 | 23 | 24 | 15 |  |
| TAPSE (mm) | 28 | 27 | 27 | 18 |  |
| IVC-D (cm) | 23 | 18 | 24 | 12 |  |
| rCBF - D (cm) | 28 | 22 | 22 | 12 |  |
| rCBF - VTI (cm) | 28 | 22 | 22 | 12 |  |
| **Calculated** |  |  |  |  |  |
| CO (l/min) | 28 | 25 | 20 | 13 |  |
| SV (ml) | 28 | 25 | 20 | 13 |  |
| IVC-CI (%) | 23 | 18 | 24 | 12 |  |
| rCBF (mL/min) | 28 | 22 | 22 | 12 |  |

This table shows the number of available image sets, divided by each parameter and observer.

Legend: LVOT-D - Left ventricular outflow tract diameter; LVOT VTI - Left ventricular outflow tract velocity time integral; EPSS – E Point septal separation; MAPSE - Mitral annular plane systolic excursion; TAPSE – Tricuspid annulus plane systolic excursion; IVC-D - Inferior vena cava diameter; rCBF-D - right Carotid blood flow diameter; rCBF-VTI - right Carotid blood flow velocity-time integral; CO – Cardiac output; SV – Stroke volume; IVC-CI - Inferior vena cava collapsibility index; rCBF – right Carotid blood flow.

* Total number of sets of images per parameter taken by all three observers that were included and used for statistical analysis

#### Supplementary Table 4: Results of image quality assessment per parameter and observer, with the total number and percentage of excluded images, missing images and total images for analysis

| **Parameter** | **Images not fulfilling the quality assessment criteria** | | | | | | | |
| --- | --- | --- | --- | --- | --- | --- | --- | --- |
|  | **Observer 1 (N=30)** | | **Observer 2 (N=27)** | | **Observer 3 (N=28)** | | **Total (N=32)** | |
| Total images taken per parameter | 90 | | 81 | | 84 | | 255 | |
| LVOT-D (cm) | 7 | 8% | 4 | 5% | 7 | 8% | 18 | 7% |
| LVOT-VTI (cm) | 8 | 9% | 9 | 11% | 27 | 32% | 44 | 17% |
| EPSS (mm) | 13 | 14% | 9 | 11% | 4 | 5% | 26 | 10% |
| MAPSE (mm) | 13 | 14% | 20 | 25% | 11 | 13% | 44 | 17% |
| TAPSE (mm) | 10 | 11% | 3 | 4% | 4 | 5% | 17 | 7% |
| IVC-D (cm) | 25 | 28% | 26 | 32% | 7 | 8% | 58 | 23% |
| rCBF measurments | 7 | 8% | 21 | 26% | 17 | 20% | 45 | 18% |
|  | **Total** | | | | | | | |
| Total images stored | 630 | | 567 | | 588 | | 1785 | |
| Total images rated inadequate | 83 | 13% | 92 | 16% | 77 | 13% | 252 | 14% |
| Total missing images | 7 | 1% | 6 | 1% | 18 | 3% | 31 | 2% |
| Total images for analysis | 540 | 86% | 469 | 83% | 493 | 84% | 1502 | 84% |

This table shows results of image quality assessment divided per parameter and observer, missing images, and total images for analysis.

Legend: LVOT-D - Left ventricular outflow tract diameter; LVOT VTI - Left ventricular outflow tract velocity time integral; EPSS – E Point septal separation; MAPSE - Mitral annular plane systolic excursion; TAPSE – Tricuspid annulus plane systolic excursion; IVC-D - Inferior vena cava diameter; rCBF-D - right Carotid blood flow diameter; rCBF-VTI - right Carotid blood flow velocity-time integral; CO – Cardiac output; SV – Stroke volume; IVC-CI - Inferior vena cava collapsibility index; rCBF – right Carotid blood flow.

#### Supplementary Table 5: Inter-researcher variability: ICC based on the comparison between operators are when all three examiners' full sets of images were available, with the 95% confidence interval and the correlating sample size number of volunteers.

| **Parameter** |  | **ICC** | **95% Confidence Interval** | | **N.** |
| --- | --- | --- | --- | --- | --- |
|  |  |  | **Lower Border** | **Upper Border** |  |
| **Measured parameters** | | | | | |
| **LVOT-D** | O1 and O2 | 0,602 | 0,260 | 0,812 | 22 |
|  | O1 and O3 | 0,694 | 0,401 | 0,858 | 23 |
|  | O2 and O3 | 0,407 | -0,014 | 0,715 | 19 |
|  | Average | 0,568 | 0,216 | 0,795 | 32 |
|  | All three operators | 0,518 | 0,237 | 0,763 | 17 |
| **LVOT-VTI** | O1 and O2 | 0,623 | 0,291 | 0,823 | 22 |
|  | O1 and O3 | 0,219 | -0,129 | 0,578 | 17 |
|  | O2 and O3 | 0,401 | -0,062 | 0,740 | 15 |
|  | Average | 0,414 | 0,033 | 0,714 | 32 |
|  | All three operators | 0,427 | 0,098 | 0,739 | 13 |
| **EPSS** | O1 and O2 | 0,566 | 0,098 | 0,815 | 20 |
|  | O1 and O3 | 0,791 | 0,567 | 0,907 | 22 |
|  | O2 and O3 | 0,363 | -0,090 | 0,696 | 21 |
|  | Average | 0,573 | 0,192 | 0,806 | 32 |
|  | All three operators | 0,567 | 0,218 | 0,810 | 16 |
| **MAPSE** | O1 and O2 | 0,457 | 0,016 | 0,746 | 20 |
|  | O1 and O3 | 0,328 | -0,111 | 0,660 | 21 |
|  | O2 and O3 | 0,440 | -0,039 | 0,749 | 18 |
|  | Average | 0,408 | -0,045 | 0,718 | 32 |
|  | All three operators | 0,409 | 0,087 | 0,714 | 15 |
| **TAPSE** | O1 and O2 | 0,274 | -0,162 | 0,615 | 23 |
|  | O1 and O3 | 0,383 | 0,009 | 0,673 | 23 |
|  | O2 and O3 | 0,363 | -0,027 | 0,668 | 22 |
|  | Average | 0,340 | -0,060 | 0,652 | 32 |
|  | All three operators | 0,303 | 0,028 | 0,605 | 18 |
| **IVC-D** | O1 and O2 | 0,772 | 0,443 | 0,918 | 15 |
|  | O1 and O3 | 0,725 | 0,372 | 0,888 | 19 |
|  | O2 and O3 | 0,568 | -0,034 | 0,852 | 14 |
|  | Average | 0,688 | 0,260 | 0,886 | 32 |
|  | All three operators | 0,655 | 0,295 | 0,876 | 12 |
| **rCBF - D** | O1 and O2 | 0,142 | -0,329 | 0,547 | 20 |
|  | O1 and O3 | 0,237 | -0,171 | 0,605 | 18 |
|  | O2 and O3 | 0,485 | -0,068 | 0,803 | 14 |
|  | Average | 0,288 | -0,189 | 0,652 | 32 |
|  | All three operators | 0,302 | -0,054 | 0,682 | 12 |
| **rCBF - VTI** | O1 and O2 | 0,094 | -0,113 | 0,385 | 20 |
|  | O1 and O3 | 0,146 | -0,206 | 0,520 | 18 |
|  | O2 and O3 | 0,093 | -0,482 | 0,589 | 14 |
|  | Average | 0,111 | -0,267 | 0,498 | 32 |
|  | All three operators | 0,152 | -0,071 | 0,511 | 12 |
| **Calculated parameters** | | | | | |
| **CO** | O1 and O2 | 0,460 | 0,046 | 0,736 | 22 |
|  | O1 and O3 | 0,612 | 0,218 | 0,837 | 17 |
|  | O2 and O3 | 0,623 | 0,179 | 0,856 | 15 |
|  | Average | 0,565 | 0,148 | 0,810 | 32 |
|  | All three operators | 0,621 | 0,309 | 0,848 | 13 |
| **SV** | O1 and O2 | 0,586 | 0,237 | 0,803 | 22 |
|  | O1 and O3 | 0,484 | 0,056 | 0,771 | 17 |
|  | O2 and O3 | 0,702 | 0,314 | 0,889 | 15 |
|  | Average | 0,591 | 0,202 | 0,821 | 32 |
|  | All three operators | 0,693 | 0,414 | 0,881 | 13 |
| **IVC-CI** | O1 and O2 | 0,418 | -0,040 | 0,748 | 15 |
|  | O1 and O3 | 0,675 | 0,326 | 0,861 | 19 |
|  | O2 and O3 | 0,415 | -0,071 | 0,759 | 14 |
|  | Average | 0,503 | 0,072 | 0,789 | 32 |
|  | All three operators | 0,472 | 0,135 | 0,777 | 12 |
| **rCBF** | O1 and O2 | -0,074 | -0,263 | 0,225 | 20 |
|  | O1 and O3 | 0,227 | -0,115 | 0,580 | 18 |
|  | O2 and O3 | 0,008 | -0,564 | 0,536 | 14 |
|  | Average | 0,054 | -0,314 | 0,447 | 32 |
|  | All three operators | 0,148 | -0,052 | 0,489 | 12 |

This table shows data about the ICC based on the comparison between operators.

Legend: LVOT-D - Left ventricular outflow tract diameter; LVOT VTI - Left ventricular outflow tract velocity time integral; EPSS – E Point septal separation; MAPSE - Mitral annular plane systolic excursion; TAPSE – Tricuspid annulus plane systolic excursion; IVC-D - Inferior vena cava diameter; rCBF-D - right Carotid blood flow diameter; rCBF-VTI - right Carotid blood flow velocity-time integral; CO – Cardiac output; SV – Stroke volume; IVC-CI - Inferior vena cava collapsibility index; rCBF – right Carotid blood flow.

### Supplementary Graphs

#### Supplementary Graph 1: Bar graph showing POCUS quantitative measurements expressed in increasing order of coefficient of variation.


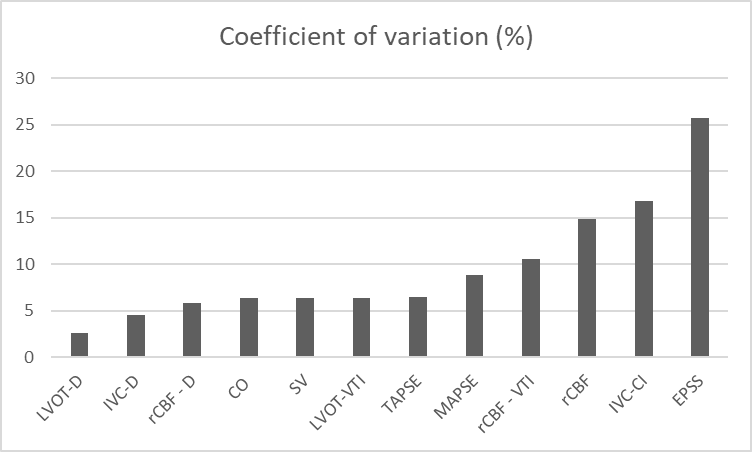


LVOT-D - Left ventricular outflow tract diameter; IVC-D - Inferior vena cava diameter; rCBF-D - right Carotid blood flow diameter; CO – Cardiac output; SV – Stroke volume; LVOT VTI - Left ventricular outflow tract velocity time integral; TAPSE – Tricuspid annulus plane systolic excursion; MAPSE - Mitral annular plane systolic excursion; rCBF-VTI - right Carotid blood flow velocity-time integral; rCBF – right Carotid blood flow; IVC-CI - Inferior vena cava collapsibility index; EPSS – E Point septal separation.
